# Additive fiber-cerclages in proximal humeral fractures stabilized by locking plates: No effect on fracture stabilization and rotator cuff function in human shoulder specimens

**DOI:** 10.3109/17453670903110659

**Published:** 2009-08-01

**Authors:** Christine Voigt, Christof Hurschler, Louise Rech, Rolf Vosshenrich, Helmut Lill

**Affiliations:** ^1^Department of Trauma and Reconstructive Surgery, Germany; ^2^Institute of MRI Diagnostics, Diakoniekrankenhaus Friederikenstift gGmbHGermany; ^3^Laboratory of Biomechanics and Biomaterials, Department of Orthopedic Surgery, Hannover Medical SchoolHannoverGermany

## Abstract

**Background and purpose** The effect of additive fiber-cerclages in proximal humeral fractures stabilized by locking plates on fracture stabilization and rotator cuff function is unclear. Here it was assessed in a human cadaver study.

**Methods** 24 paired human shoulder specimens were harvested from median 77-year-old (range 66–85) female donors. An unstable 3-part fracture model with an intact rotator cuff was developed. 1 specimen of each pair received an additive fiber-cerclage of the rotator cuff after plate fixation, and the other one received a plate fixation without an additive fiber-cerclage. Force-controlled hydraulic cylinders were used to simulate physiological rotator cuff tension, while a robot-assisted shoulder simulator performed 4 relevant cases of load: (1) axial loading at 0°, (2) glenohumeral abduction at 60°, (3) internal rotation at 0° abduction, and (4) external rotation at 0° abduction, and imitated hanging arm weight during loading without affecting joint kinematics. A 3-dimensional real-time interfragmentary motion analysis was done in fracture gaps between the greater tuberosity and the head, as well as subcapital. The capacity of the rotator cuff to strain was analyzed with an optical system.

**Results** Interfragmentary motion was similar between the groups with and without fiber-cerclages, in both fracture gaps and in any of the cases of load. Cerclages did not impair the capacity of the rotator cuff to strain.

**Interpretation** Provided that unstable 3-part fractures are reduced and stabilized anatomically by a locking plate, additive fiber-cerclages do not reduce interfragmentary motion. Additive fiber-cerclages may be necessary in locking plate osteosyntheses of multiple-fractured greater tuberosities or lesser tuberosity fractures that cannot be fixed sufficiently by the plate.

## Introduction

Locking plates have become a standard, well-established surgical treatment for displaced proximal humeral fractures. However, the goal of an anatomical reduction and stable retention until fracture healing occurs is not achieved in all patients. Secondary displacement or secondary head deformities have been reported in more than 20% of elderly patients (Fankhauser et al. 2005, Kettler et al. 2006, Voigt et al. 2007). Typical failure modes are caused by rotator-cuff forces (Gardner et al. 2007). Consequently, it was postulated that fixation constructs should be augmented, as necessary, with sutures passing adjacent to bony fragments through rotator cuff tissue and back to the fixation implant, to provide maximum implant-fragment stability (Smith et al. 2007). Additive fiber-cerclages should reduce displacing forces of the rotator cuff (Hente et al. 2004, Fankhauser et al. 2005, Voigt et al. 2007), and secure tuberosities after reduction in their position as a stable circular platform on which the head fragment can rest (Hertel 2005, Nho et al. 2007). Casustically, in angle stable plate fixations without additive fiber-cerclage, subacromial impingement and tuberosity displacement were observed more frequently than with an additive fiber-cerclage (Hente et al. 2004).

The purpose of this biomechanical study was to investigate the effect of the clinically established but still contentiously discussed additive fiber-cerclages in proximal humeral fractures stabilized by locking plates, on fracture stabilization and rotator cuff function. The hypothesis was that an additive fiber-cerclage reduces interfragmentary motion in an unstable 3-part fracture model stabilized by a locking plate.

## Materials and methods

### Specimen preparation

26 paired female fresh-frozen human specimens (Science Care, Phoenix, AZ) from donors with a median age of 77 (66–85) years and without any known history of shoulder pathology were investigated. Median body mass index (BMI) was 25 (13–43). Anteroposterior (AP) and axillary views were examined to preclude fractures or other osteopathologies. Dual X-ray absorptiometry (DXA) of all humeral heads was performed using a modified scan-mode (Discovery QDR for Windows; Hologic, Bedford, MA) which allowed free definition of 6 regions of interest (ROIs) (crosswise: proximal, central, distal, shaft, medial, and lateral) in the humeral head (Lill et al. 2002). The average bone mineral density (BMD) of the humeral heads investigated was 0.434 (SD 0.191) g/cm^2^. Shoulder specimens of osteoporotic women (> 65 years) were intentionally selected since they represent a high-risk group for proximal humeral fractures with typical complications such as secondary fragment displacement and varus deformity.

The integrity of the rotator cuff of all specimens was assessed by MRI. 2 complete supraspinatus tendon tears were observed, and these shoulders were excluded from the study. 2 other specimens revealed partial supraspinatus tendon tears from the bursa side (≤ 5 mm), which were adapted by sutures. 4 other specimens had a partial supraspinatus tendon tear from the articular side, visible by MRI. 3 pairs of specimens had a muscular atrophy of M. supraspinatus (SSP) Thomazeau I°, 1 pair Thomazeau I° (right) and II° (left), 7 pairs Thomazeau II° (Thomazeau et al. 1996). 2 left shoulders were paired because of a complete rotator cuff tear of the contralateral shoulder, and had SSP atrophy Thomazeau II° and III°. No M. infraspinatus (ISP) or M. subscapularis (SSC) tears observed by MRI or macroscopically.

Specimens were frozen to –20° for storage and subsequently thawed at room temperature for 12 h. During dissection, preparation, and testing, the specimens were moistened with physiological saline solution to prevent dehydration. Soft tissues superficial to the rotator cuff were removed, leaving the scapula with attached SSP, M. infraspinatus and M. teres minor (ISP+TM) and SSC, as well as the joint capsule with its associated ligaments intact. The clavicula was transected at half-shaft, the humerus approximately 20 cm from the center of the humeral head. The rotator cuff tendons were bluntly separated from the surrounding tissue to ensure unrestricted movement.

### Fracture model

**Figure 1.**
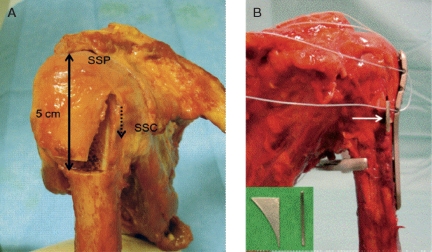
A. Right shoulder: osteotomy of the greater tuberosity. SSP: M. supraspinatus; SSC: M. subscapularis; …..> bicipital groove. B. Placeholder (superimposed display and arrow) in gap l to retain a 2-mm gap between the head and greater tuberosity fragment during plate fixation.

An unstable 3-part fracture model with an intact rotator cuff was developed to simulate (as physiologically as possible) unstable 3-part fractures, which are the most common fractures in the elderly (Voigt et al. 2007). Thus, an osteotomy of the greater tuberosity was performed starting 5 cm distal to the top of the humeral head and ventral directly lateral to the bicipital groove. The bony greater tuberosity, including the insertion zones of SSP and ISP+TM, was completely separated without cutting through the cranially and dorsally inserting SSP and ISP+TM tendons (Figure 1a).

Specially designed 2-mm wide metal blades were placed ventrally and dorsally in the osteotomy fissure, to act as a placeholder and affect a 2-mm wide gap (gap I) (Figure 1b).

An osteosynthesis was subsequently performed using a Proximal Humerus Internal Locking System (PHILOS; Synthes, Umbirch, Germany) in 6 specimen pairs (right and left shoulder of the same donor), and a humeral suture plate (HSP; Arthrex, Karlsfeld, Germany) in 6 other pairs. The plates were placed anatomically, and were attached with 6 angle stable head screws and 3 shaft screws, respectively. Humeral-head screws were placed close to the medial cortical bone. Subsequently, a subcapital distance osteotomy (height: 5 mm) at the level of the surgical neck was performed 5 cm distal to the top of the head (gap II) (Lill et al. 2001).

**Figure 2.**
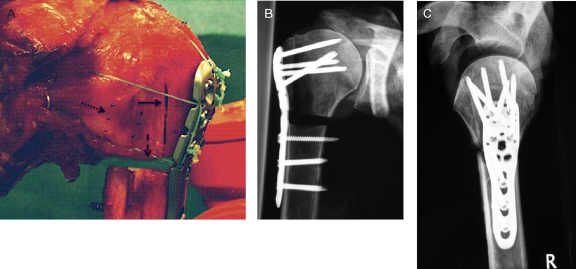
A. Unstable 3-part fracture model with fiber-cerclages of the proximal humerus intact rotator cuff. Fracture gap I (à) and II (- ->). Three point-pairs (….>) for measurement of rotator cuff strain. B. AP and C. axillary views of an unstable 3-part-fracture model.

All left shoulders of the pairs received a standardized additive fiber-cerclage of SSC, SSP, and ISP+TM (Figure 2a). Thus, 3 non-absorbable Fiber-wire size 2 (Arthrex) were pre-placed in specially designed plate holes. The SSC was attached by a 2-cm wide u-shaped stitch 4 cm away from the ventral edge of the plate, SSP, and ISP+TM by diverging 2-cm wide u-shaped stitches 2 cm away from the cranial and dorsal edges, respectively. Sutures were knotted and the metal blades in gap I excluded. All specimens were examined radiographically after preparation to control and document correct implant and gap positioning (Figure 2b).

In preparation for attachment of cables for application of forces to the muscles, the muscles were first elevated from the scapula. The rotator cuff was divided. The border between SSC and SSP was the rotator interval; the border between SSP and ISP+TM was defined to be in line with the spine of the scapula. Ductile brass wires were woven into the musculotendinous junctions of SSC, SSP, and ISP+TM using Kirchmayer-Kessler technique.

### Testing device

The distal humeral shaft was potted in a brass cylinder (diameter: 38 mm; height: 50 mm) using a methylmethacrylate resin. The axis of the humerus was defined by aligning the humerus and the cylinder using a jig inserted in the marrow cavity.

The scapula was aligned and rigidly mounted in the testing device using 3 threaded steel rods placed by a standardized procedure, so that the medial margin of the scapula was in line with the vertical axis of the device. The scapula was tilted forward 10° to approximate its physiological orientation on the thorax. The humerus shaft was fixed to the robot-assisted shoulder simulator (RASS) (KUKA GmbH, Augsburg, Germany) (Klages et al. 2001) by inserting the brass cylinder into a mount attached to the force-moment sensor. Neutral rotation of the humerus was defined by alignment of the intertubercular groove at the humeral head, midway between the coracoid process and the lateral margin of the acromion.

**Figure 3.**
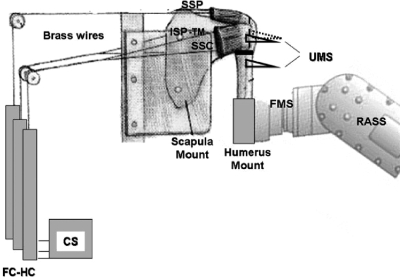
The experimental setup. SSP: M. supraspinatus; ISP-TM: M. infraspinatus and m. teres minor; SSC: M. subscapularis; UMS: ultrasonic measuring system; FMS: force moment sensor; RASS: robot-assisted shoulder simulator; FC-HC: force-controlled hydraulic cylinder; CS: control station.

SSC, SSP, and ISP+TM were each connected to force-controlled hydraulic cylinders via brass wires sutured to the respective muscle insertion. Each hydraulic cylinder (AHS-25/G 128; Fa GHS, Ilsfeld-Auenstein, Germany) was regulated by means of a force sensor (Fa Megatron, Putzbrunn, Germany) attached close to the muscle (Figure 3).

To simulate physiological tension of the rotator cuff in the neutral shoulder position, muscles were loaded along their physiological line of action, proportionally to their respective cross-sectional areas: 66 N was applied to SSC, 26 N to SSP, and 61 N to ISP+TM (Veeger et al. 1991, Halder et al. 2002)

### Testing sequence

4 clinically relevant loads were applied using the hydraulic system and RASS: (1) axial load (120 N) at 0° of glenohumeral abduction, (2) axial load at 60° of glenohumeral abduction, (3) internal rotation at 0° abduction, and (4) external rotation at 0° abduction.

During specimen loading, rotator-cuff muscle forces were applied using the force-controlled hydraulic cylinders, while the RASS simulated axial loading (120 N) in modes 1 and 2, and resisted rotation in modes 3 and 4. During modes 1 and 2, all the muscles of the rotator cuff received their physiological tensions in neutral joint position. In the internal rotation mode (3), the SSC was loaded with its physiological tension, and SSP and ISP+TM were loaded to 10 N to simulate co-contraction; in external rotation mode 4, the ISP+TM was loaded with its physiological tension, whereas the SSC and SSP were loaded to 10 N.

Interfragmentary motion in gaps I and II was assessed with an ultrasonic 3-dimensional (3D) motion analysis system (CMS 20S; Zebris Medical GmbH, Isny, Germany) with a spatial resolution of ± 0.1 mm (Figure 3). In measuring motion, head fragment motion was measured relative to the greater tuberosity for gap I, or to the shaft for gap II. A 3D coordinate system was defined distal to the angle stable plate at the humeral shaft using the motion analysis software (WinBioMechanics Version 0.1.2; Zebris Medical), where the anterior-posterior direction (y-axis) was normal to the scapular plane, the inferior-superior (z-axis) was vertical in the scapular plane, and medial-lateral (x-axis) was orthogonal to the y- and z-axes. 2 defined reference points medial and lateral to the distal edge of gap I, or superior and inferior to gap II (lateral edge), were also defined.

Fragment motion was measured between the unloaded and loaded states in all specimens and all loading modes at gap I and gap II.

### Measurement of rotator cuff strain

Changes in muscle insertion strain due to fiber-cerclage were analyzed with an optical system. On each muscle, 3 point-pairs (< 1 mm) were marked with molybdenum graphite at a distance of 0.5 cm besides the 2-cm mark distal from the plate's edge in the direction of muscle traction (Figure 1a). A digital image was taken under a 10 N loading of SSC, SSP, and ISP+TM, and a second one was taken after applying the physiological load to each muscle. Changes in distance between the markers were analyzed using image analysis software (NIH-ImageJ; W. Rasband, NIH, Bethesda, MD).

### Statistics

All data are reported as mean (SD) and were analyzed by means of descriptive statistics using SPSS software version 14.0. Power analysis (statistical significance, α = 5%; type-II error, β = 20%; power, (1 – β) = 80%; and magnitude of difference = 0.3 mm) was performed to determine the minimum sample size (n = 6). All statistical tests were applied two-sidely and non-parametrically because analysis of the data did not show a normal distribution. The Wilcoxon test was performed to compare related samples. All p-values ≤ 0.05 were considered statistically significant. Mixed-model analysis of variance (ANOVA) was performed to detect differences in gap motion dependent on surgical technique.

## Results

Matched-pair analysis showed no statistically significant differences in 3-dimensional total interfragmentary motions in gap I or II between the groups with (n = 12) and without (n = 12) additive fiber-cerclages of the SSC, SSP, and ISP+TM in (1) axial load at 0° glenohumeral abduction, (2) axial load at 60° glenohumeral abduction, (3) internal rotation at 0° abduction, and (4) external rotation at 0° abduction (Table 1).

**Table 1.** Three-dimensional motions in gap I and II in four physiological-like load cases with (n = 12) and without (n = 12) additive fiber-cerclage. Mean (SD) in mm

	Axial load in 0° abduction	Axial load in 60° abduction	Internal rotation	External rotation
Gap I				
with cerclage	0.29 (0.25)	0.33 (0.22)	0.19 (0.23)	0.21 (0.18)
without cerclage	0.34 (0.28)	0.34 (0.30)	0.20 (0.15)	0.19 (0.17)
p-value	0.5	0.7	0.5	0.9
Gap II				
with cerclage	1.16 (1.20)	0.91 (0.84)	0.38 (0.37)	0.62 (0.68)
without cerclage	1.12 (0.83)	0.75 (0.61)	0.49 (0.41)	0.31 (0.23)
p-value	1.0	0.7	0.3	0.3

The largest 3-dimensional interfragmentary motion was found in mode 1 to be 0.34 mm in gap I, and 1.16 mm in gap II, respectively. There was no significant difference in interfragmentary motion between the 4 different load cases in gap I and gap II. Motion in gap II was statistically significantly larger than in gap I (Table 1). The fracture model was elastic, non-persisting displacement of tuberosity or head fragment was observed.

Separately viewing the components of motion, modes 1 and 2 had the greatest effect on vertical motion (z-axis motion). There was no significant difference in z-axis motion with and without cerclage in gaps I and II (Table 2).

**Table 2.** Vertical motions (z-axis motions) in physiological-like load cases with (n = 12) and without (n = 12) additive fiber-cerclage. Mean (SD) in mm

	Axial load in 0° abduction	Axial load in 60° abduction
GAP I		
with cerclage	0.79 (0.99)	0.34 (0.28)
without cerclage	0.53 (0.65)	0.42 (0.29)
p-value	0.4	0.5
GAP II		
with cerclage	2.12 (1.37)	1.06 (0.83)
without cerclage	1.24 (0.92)	0.75 (0.45)
p-value	0.05	0.5

Vertical motion was greater in gap II than in gap I in mode 1 (p = 0.001) and in mode 2 (p = 0.002); and in gap II larger in mode 1 than in mode 2 (p = 0.02).

**Figure 4.**
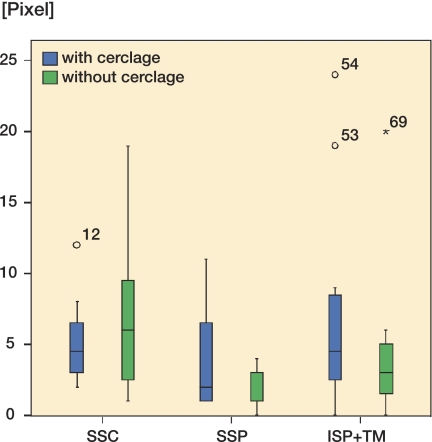
Measurement of rotator cuff strain with (n = 12) and without (n = 12) additive fiber-cerclage. SSC: M. subscapularis; SSP: M. supraspinatus; ISP+TM: M. infraspinatus and m. teres minor.

The capacity of SSC, SSP, and ISP+TM to contract was not statistically significantly impaired (p = 0.5, p = 0.1, and p = 0.2) by an additive fiber-cerclage under physiological tension of the rotator cuff (Figure 4).

Mixed-model ANOVA did not show any significant influence of different surgical techniques (with/without fiber-cerclage) on the gap widths measured.

## Discussion

This study was a real-time analysis of interfragmentary motion by using a newly developed physiological fracture model with an intact rotator cuff, which represents the most frequent fracture type of the proximal humerus in the elderly (Fankhauser et al. 2005, Agudelo et al. 2007, Voigt et al. 2007). With this model, we assessed the effect of additive fiber-cerclages in proximal humeral fractures stabilized by locking plates—in terms of the fragment dislocation behavior in the early postoperative period. This distinguishes our study from previous studies that analyzed isolated bone models in cyclical testings to evaluate bone-implant-interface failure and implant failure (Lill et al. 2001, Edwards et al. 2006, Walsh et al. 2006, Sanders et al. 2007).

Here, a robot-assisted shoulder simulator allowed defined active motion and loading by imitating the hanging arm weight in each position without affecting joint kinematics (Klages et al. 2001, Kedgley et al. 2007). The loadings chosen simulated physiological situations such as axial loading at 0° abduction, while “the first getting up” of the elderly patients after surgery, or 60° glenohumeral abduction, which is equal to an arm abduction of about 90° (Poppen and Walker 1976), and internal and external rotations.

This setup could not confirm our hypothesis that an additive fiber-cerclage would reduce interfragmentary motion in an unstable 3-part fracture model stabilized by a locking plate.

The specimens used in this investigation were chosen from female donors who were more than 65 years old, because they represent a high-risk group for sustaining proximal humeral fractures and postoperative complications such as secondary fragment displacement and deformities (Hente et al. 2004, Fankhauser et al. 2005, Voigt et al. 2007). The BMD of the specimens was found to be similar to values reported for patients of similar age and sex (Lill et al. 2002, Hepp et al. 2003). The quality of rotator cuffs observed, with 2/26 complete and 6/26 partial SSP tears, was to be expected in this selection of donors (Nanda et al. 2007). An increasing prevalence of rotator cuff tears with advancing age and a 50% likelihood of a bilateral tear after the age of 66 years have been reported (Yamaguchi et al. 2006).

Mechanical studies of proximal humeral fractures have mainly been performed with respect to the bony structures (Ruch et al. 2000, Lill et al. 2001, Edwards et al. 2006, Sanders et al. 2007), but retention after reduction essentially depends on the insertion of the rotator cuff working as the main dislocator of the fragments. In the only previously described study to preserve the rotator cuff in a 2-part fracture model of the humeral head, rotator cuff muscles were loaded unphysiologically by clamping them together in one direction to a metal cylinder, which was fixed cranially in a materials-testing machine in order to analyze implant failure (Walsh et al. 2006).

Our fracture model involves the complex anatomy of the humeral head, by preserving and physiologically loading the intact rotator cuff. It was achieved by creating a reproducible, unstable 3-part facture model in human shoulder specimens. 3-part fractures are the commonest fracture type in elderly, osteoporotic people and they have a high complication rate (Hente et al. 2004, Voigt et al. 2007). Previously, mechanical studies of proximal humeral fractures mainly used 2-part models with subcapital distance osteotomies of 5–10 mm (Lill et al. 2002, Edwards et al. 2006, Walsh et al. 2006). Rarely described 3-part models were synthetic, or were not unstable in the fracture line between the greater tuberosity and the humeral head (Ruch et al. 2000, Sanders et al. 2007).

Fiber-cerclage, applied in proximal humeral fractures stabilized by locking plates to further secure tuberosity fragments after reduction, is often used to minimize secondary fragment displacement (Nho et al. 2007, Voigt et al. 2007). Casustically, more frequently, tuberosity displacement has been observed in locked-plate fixations without additive fiber-cerclage (Hente et al. 2004). We have found no clinical or biomechanical studies about the effects of additive fiber-cerclages.

We observed no effect of additive fiber-cerclages of the rotator cuff on interfragmentary motion in osteoporotic bone. In the case of anatomical reduction of such a 3-part-fracture, when the main greater tuberosity fragment is sufficiently fixed and completely covered by the plate, additive fiber-cerclages are not required.

It is, however, important to point out that in real fracture surgery additive fiber-cerclages may still be essential for tuberosity reduction, and may be useful to improve stability in cases of suboptimal reduction, multiple fractures, or only partially plate-covered greater tuberosity fragments by retaining them near their anatomical position, and reducing displacement forces (Hente et al. 2004, Walsh et al. 2006, Voigt et al. 2007). In 4-part fractures, additional fiber-cerclages stabilize lesser tuberosity fragments that cannot obtain stability from the plate (Hente et al. 2004, Walsh et al. 2006).

In the defined, intraoperatively feasible dimension, fiber-cerclages did not reduce rotator cuff function. The statistically non-significant trend toward a little more elongation in SSP and ISP+TM in the group with cerclage can be explained by a slight folding developed during performance of fiber-cerclages. By physiological loading of the rotator cuff, folding was removed, and caused non-significantly more changes of length in SSP and ISP+TM. This effect could have been captured by measuring the length before cerclaging.

The strengths of our study are the comparatively high number of specimens per group, the unstable 3-part fracture model developed with an intact rotator cuff, the real-time analysis of interfragmentary motion under physiological loading, and the use of a robot with specially written software for fracture-model testing. The use of paired specimens permitted a matched-pair analysis with comparable results between the groups.

One limitation of our study is the fracture model, which is on the one hand more physiological than previous models and a progression to the unstable 2-part models, but on the other hand it still cannot imitate multiple-fractured, small-tuberosity fragments often seen in the elderly (Voigt et al. 2007, Hertel 2005). The complex and time-consuming experimental setup, which allows real-time analysis, is not suitable for cyclical testing. Further studies will be required to analyze additive fiber-cerclages in proximal humeral fractures stabilized by locking plates, in long-term loading studies and in randomized prospective clinical studies.
